# Identified barriers and facilitators to stroke risk screening in children with sickle cell anemia: results from the DISPLACE consortium

**DOI:** 10.1186/s43058-021-00192-z

**Published:** 2021-08-10

**Authors:** Shannon M. Phillips, Alyssa M. Schlenz, Martina Mueller, Cathy L. Melvin, Robert J. Adams, Julie Kanter

**Affiliations:** 1 College of Nursing, Medical University of South Carolina, 99 Jonathan Lucas St, Charleston, USA; 2 Department of Pediatrics, Medical University of South Carolina, Charleston, USA; 3 Department of Pediatrics, University of Colorado School of Medicine, 1800 Grant St. Suite 800, Denver, CO 80203 USA; 4 Department of Public Health Sciences, Medical University of South Carolina, Charleston, USA; 5 Department of Neurology, Medical University of South Carolina, Charleston, SC 29425 USA; 6 Division of Hematology & Oncology, University of Alabama at Birmingham, 1720 2nd Ave. South, Birmingham, AL 35294 USA

**Keywords:** Sickle cell anemia, Qualitative, Transcranial Doppler screening, Children, Caregivers, Healthcare providers, Social ecological model

## Abstract

**Background:**

Children with sickle cell anemia are at risk for stroke. Ischemic stroke risk can be identified among children ages 2–16 years with sickle cell anemia using transcranial Doppler ultrasound. Despite strong recommendations for transcranial Doppler screening in guidelines released by the National Heart, Lung, and Blood Institute, implementation of transcranial Doppler screening in sickle cell anemia remains suboptimal. The purpose of this study was to identify barriers and facilitators to transcranial Doppler screening in a large national consortium to inform subsequent implementation interventions.

**Methods:**

A qualitative descriptive approach was used to conduct 52 semi-structured interviews with a sample of patients with sickle cell anemia, their parents or primary caregivers, and healthcare providers dispersed across the United States. Interviews took place from September 2018 through March 2019. Directed content analysis was conducted with an adapted version of the Multilevel Ecological Model of Health as an initial coding framework, completed July 2019. Frequency analysis was conducted to determine predominant barrier and facilitator themes.

**Results:**

Fourteen barrier themes and 12 facilitator themes emerged representing all levels of the ecological framework. Two barrier themes (*Logistical Difficulties and Competing Life Demands* and *Gaps in Scheduling and Coordination*), and 5 facilitator themes (*Coordination, Scheduling and Reminders*; *Education and Information*; *Provider and Staff Investment and Assistance*; *Positive Patient Experience*; and *Convenient Location*) were predominant.

**Conclusions:**

Barriers and facilitators to transcranial Doppler screening in children with sickle cell anemia are complex and occur across multiple ecological levels. One barrier theme and 3 facilitator themes were found to be optimal to address in subsequent implementation interventions.

**Supplementary Information:**

The online version contains supplementary material available at 10.1186/s43058-021-00192-z.

Contributions to the Literature

- Implementation of stroke prevention guidelines in sickle cell anemia is suboptimal.
- Barriers to the implementation of guidelines are complex, necessitating a comprehensive, ecological assessment of challenges to implementing guidelines.
- The barriers and facilitators identified inform the design of implementation interventions that are adaptable and can be applied in settings with differing characteristics.

## Background

Stroke prevention is a critical component of care for children with sickle cell anemia (SCA), an inherited blood disorder characterized by abnormal hemoglobin resulting in intermittent blood vessel occlusion and multi-organ dysfunction. Without intervention, there is an estimated 11% chance that a child with SCA will have an ischemic stroke by age 20 [1]. Stroke prevention practices for SCA were dramatically transformed by the Stroke Prevention Trial in Sickle Cell Anemia (STOP) and Optimizing Primary Stroke Prevention in Sickle Cell Anemia (STOP II) studies. These trials demonstrated children ages 2–16 years with SCA at high-stroke risk could be identified using transcranial Doppler (TCD), an ultrasound that measures blood vessel velocity in the cerebral arteries. For children with abnormally high TCD velocities in the STOP trials, the stroke rate was substantially reduced by initiating chronic red cell transfusion (CRCT) therapy [2, 3]. Adoption of the STOP protocols led to reductions in the rate of ischemic stroke in children with SCA [4–10] and in the disparity between African American and White children at risk for ischemic stroke-related death [11]. On the basis of the STOP studies, the 2014 National, Heart, Lung, and Blood Institute (NHLBI) guidelines and the 2020 American Society of Hematology (ASH) guidelines formally adopted annual TCD screening and CRCT initiation as practices for reducing stroke risk in children with SCA. These guidelines specify that children should receive annual TCD screenings from the ages of 2 to 16, with referral to a specialist in CRCT for those with abnormal screenings [12–14].

Despite these advances in care, the implementation of TCD screening remains suboptimal. A follow-up study to the STOP trials (Post STOP) demonstrated wide variation in TCD screening rates, even among sites that participated in the original stroke prevention trials; similar results have been found in smaller-scale studies [15–18]. Reasons for variation are likely multifactorial, occurring at the intersection of multiple ecological levels, including the patient (e.g., child age; caregiver knowledge, education, and self-efficacy; extent of involvement with the health care system), provider (e.g., provider knowledge, self-efficacy, and expectations), and organizational/systems (e.g., access to TCD, insurance status) levels [15, 16, 18–26].

To improve the implementation of stroke prevention practices for children with SCA, the Dissemination and Implementation of Stroke Prevention Looking at the Care Environment (DISPLACE) study was developed. DISPLACE was designed to evaluate the current implementation of stroke prevention practices (part 1), assess barriers and facilitators to stroke prevention practices (part 2), and design and deliver interventions to improve the implementation of stroke prevention practices for children with SCA (part 3). The DISPLACE consortium consists of 28 sites dispersed across the U.S. in areas with concentrated populations of individuals with SCA. Each site had a pediatric hematology/oncology department for specialized care of individuals with SCA. Sites varied on size, number of patients, geographic region, and prior participation in stroke prevention trials. In part 1 of DISPLACE, implementation rates among the 28 consortium sites ranged from 30.9 to 74.7% of children with SCA receiving annual screening as per NHLBI guidelines [27]. The purpose of this paper is to describe results from part 2 of DISPLACE, an assessment of barriers and facilitators to TCD screening from provider and patient and parent/primary caregiver perspectives. This component involved a comprehensive, ecological analysis of barriers and facilitators that could broadly inform care and a focused analysis of remediable factors to inform specific interventions for the implementation study in part 3 of the project.

## Methods

### Participants and recruitment

Semi-structured interviews were conducted with patients with SCA or the parent/primary caregiver (subsequently referred to as “caregiver”) and healthcare providers of patients with SCA using a qualitative descriptive approach [28]. Purposive sampling was used to identify patient/caregiver participants who had experienced TCD screening (both on schedule and overdue), received normal and abnormal TCD screening results, and were familiar with CRCT therapy. Patients were intentionally identified by providers within the DISPLACE consortium to ensure a wide representation of disease severity, socioeconomic, and history of adherence characteristics (e.g., attended TCD and clinic appointments in the last year vs. missed TCD and 2 or more clinic appointments in the last year) in a nationally dispersed sample. Once potential participants were identified, permission to share contact information with the authors was obtained by study personnel at individual DISPLACE consortium sites. Provider participants were identified using purposive and snowball sampling strategies to obtain a range of specialties, disciplines, and levels of experience (i.e., years providing care for patients with SCD) in a nationally dispersed sample, including providers not part of the consortium. Participants were approached by phone or email; all participants who responded to the phone call or email from interviewers consented to participate in the study, and no participants dropped out.

### Interview guide

Semi-structured interview guides with open-ended questions were developed for patients/caregivers and providers using the Multilevel Ecological Model of Health [29] as a lens to assess multi-level barriers and facilitators to care for children with SCA. This model was selected to guide interviews and analysis because a broad lens was needed to determine barriers and facilitators at the individual, organizational, and societal levels, including the interactions between individuals and the care environment [30–32]. Patient/caregiver interview guides included five main sections (Supplement 1), and provider interview guides included six main sections (Supplement 2). The sections on TCD were the focus of this analysis. The interview guide was not pilot-tested; however, it was adapted from an interview guide used in a prior study of barriers and facilitators to care in individuals with SCD [33, 34].

### Procedures

Interviews were conducted September 2018 through March 2019 by SP, a female PhD-prepared nurse and AS, a female PhD-prepared psychologist. Both SP and AS have training and experience in qualitative research methods, data collection, and analysis, and experience in the care of individuals with sickle cell disease. Informed consent was obtained from all participants before demographic data were collected and the interview commenced. Prior to obtaining consent, the interviewers described the purpose of the study and their backgrounds and role in the study. Institutional Review Board approval was obtained prior to data collection.

Interviews were conducted in person or by phone. In-person interviews were conducted in a private room at a sickle cell clinic with only the interviewer and participant present. Interviewers conducted phone interviews from a private office with the door closed and advised participants in advance to be in a private location at the time of the interview. Following the interview, patient/caregiver participants received compensation of a $100 gift card. Provider participants did not receive compensation. Interviews were audio-recorded, lasted a range of 30 to 75 min, and were transcribed for analysis. Field notes were recorded during and after the interviews and were discussed by the interviewers following each interview. Data saturation was reached at the 27th patient/caregiver interview and the 25th provider interview for a total of 52 interviews. No repeat interviews were conducted, and transcriptions were not returned to participants for comment.

### Data analysis

Data analysis began after the first interview and was completed in July 2019. Directed content analysis [35] was conducted using a codebook created with an adaptation of the Multilevel Ecological Model of Health as an initial coding framework and a deductive-inductive approach. First, high-level codes were created deductively to represent ecological levels, and subsequently, subcodes within the high-level codes were developed inductively to represent barriers and facilitators within each ecological level. The constant comparison method [36] was used to place data into codes. Analysis was conducted using Microsoft Word documents and Excel spreadsheets. Each transcript was coded by SP and AS, and in cases of disagreement, decisions were discussed until the resolution was reached. Provider and patient/caregiver transcripts were analyzed separately. After themes were developed for each group, patient/caregiver and provider themes and coding definitions were compared to identify convergence and divergence and were organized by ecological level, including patient, provider, organizational, and social-environmental and policy levels. Frequency analysis was conducted to identify predominant barriers and facilitators to inform interventions in the next phase of the study. The number of participants (patients/caregivers and providers) who contributed data to each theme was tallied. Themes with at least 20% (or 10 participants) representation were considered predominant.

To establish priorities for addressing barriers or enhancing facilitators, and to maximize resource allocation [31], predominant themes were assessed for remediability. The goal was to identify interventions with the potential for addressing as many barriers and facilitators as possible while considering challenges, such as diverse practice settings and resource limitations. Predominant themes were first reviewed and discussed by the study team to determine whether addressing each theme was likely to yield an improvement in the TCD implementation rate (the primary outcome in the next phase of the study). We also considered whether themes were more evenly represented in terms of patient/caregiver and provider perceptions. Then, to provide structure to setting priorities and to assist with decision making, relevant intervention characteristic concepts from the Consolidated Framework for Implementation Research (CFIR) [37] were applied, specifically, cost, complexity, and adaptability. Interventions needed to be low-to-moderate cost because of resource limitations, highly adaptable because of diversity in practice settings, and low-to-moderate complexity for successful implementation in the context of a multicomponent intervention. Further, the CFIR was used to inform part 3 of the DISPLACE project; CFIR constructs were applied during the remediability assessment to promote continuity from determining interventions to the evaluation of the implementation context. The central study team with expertise in medicine, nursing, psychology, and implementation science discussed findings and potential interventions, and assessed potential interventions for remediability to determine which predominant barriers to address and facilitators to adopt. Discussions also took place with the DISPLACE site principal investigators during all-site conference calls. The Consolidated criteria for Reporting Qualitative research (COREQ) checklist was used to develop this manuscript [38].

## Results

Characteristics of participants are presented in Tables 1 and 2. Patient/caregiver interviews were conducted primarily with caregivers of affected children; the majority were female and African American. Patient/caregiver participants resided in 15 states across the U.S. representing regions with concentrated populations of individuals with SCA. Provider participants were primarily White, physicians, and practicing in pediatric hematology/oncology in 16 states across the U.S.

**Table 1:** Patient and caregiver demographics

**Individual with sickle cell anemia (*n*= 27)**		**Caregiver (*n*= 26)**	
Age (years)	*M* = 11.6, median = 12 (range 3–18)	Age (years)	*M* = 39.0, median 39 (range 26–52)
Gender	19 (70.4%) female	Gender	22 (84.6%) female
Race/ethnicity	26 (96.3%) African American 1 (3.7%) White 1 (3.7%) Hispanic/Latino 1 (3.7%) other	Race/ethnicity	24 (92.3%) African American 2 (7.7%) White 1 (3.7%) Hispanic/Latino
Sickle Cell Genotype	25 (92.6%) HbSS 1 (3.7%) HbSß^0^ thalassemia 1 (3.7%) don’t know	Relationship to individual with SCA	21 (80.8%) mother 4 (15.4%) father 1 (3.8%) aunt
Insurance	21 (77.8%) Medicaid 5 (18.5%) private 2 (7.4%) government program 1 (3.7%) Medicare 1 (3.7%) SCHIP	Education	3 (11.5%) less than high school 11 (42.3%) high school degree/GED 3 (11.5%) some college 9 (34.6%) college degree
Healthcare utilization (last year) Hospitalizations ED visits Doctor’s office visits	*M* = 2.6, median = 2 (range 0–15) *M* = 3.5, median = 3 (range 0 – 12) *M* = 10.2, median = 8 (range 2–48)	Employment status	20 (76.9%) working 3 (11.5%) temporarily unemployed 2 (7.7%) disabled 1 (3.8%) keeping house
State of residence	1 Arkansas 3 Colorado 2 Delaware 2 Florida 1 Georgia 1 Illinois 1 Indiana 1 Maryland 1 Mississippi 3 Missouri 2 New Jersey 2 Ohio 4 South Carolina 1 Texas 2 Washington, DC	Marital status	11 (42.3%) single, never married 8 (30.8%) married 3 (11.5%) divorced 1 (3.8%) cohabitating 1 (3.8%) widowed 1 (3.8%) prefer not to respond 1 (3.8%) other
		Annual household income	8 (30.8%) less than $20,000 8 (30.8%) $20,000–$39,000 2 (7.7%) $40,000–$59, 000 2 (7.7%) $60,000–$79,000 2 (7.7%) $80,000–$94,999 1 (3.8%) $95,000 and over 2 (7.7%) prefer not to respond 1 (3.8%) missing

*SCA* sickle cell anemia, *GED* general education diploma, *ED* emergency department

**Table 2:** Provider demographics (*n* = 25)

**Age (years)**	*M* = 50.0, median 50 (range 36–66)	
**Gender**	14 (56%) female	
**Race/ethnicity**	20 (80%) White 3 (12%) African American 2 (8%) Asian 1 (4%) Hispanic/Latino	
**Provider type**	19 (76%) MD 2 (4%) NP 1 (4%) PA 3 (12%) other	
**Area of practice**	14 (56%) Hematologist (sickle cell-specific) 7 (28%) Hematologist/oncologist 4 (16%) Pediatrics	
**Years providing care to patients with sickle cell disease**	*M* = 18.8, median 17 (range 5–44)	
**Age range of patients**	25 (100%) infancy through adolescence 22 (80%) young adult 4 (16%) adult	
**Practice setting**	21 (84%) urban 5 (20%) suburban 1 (4%) rural	
**State of practice**	1 California 1 Colorado 2 Florida 2 Georgia 2 Illinois 1 Indiana 1 Louisiana 1 New Jersey	1 Massachusetts 1 Mississippi 4 Missouri 1 New York 3 North Carolina 2 South Carolina 1 Virginia 1 Washington DC

*MD* Doctor of Medicine, *NP* nurse practitioner, *PA* physician assistant

**Fig. 1:**
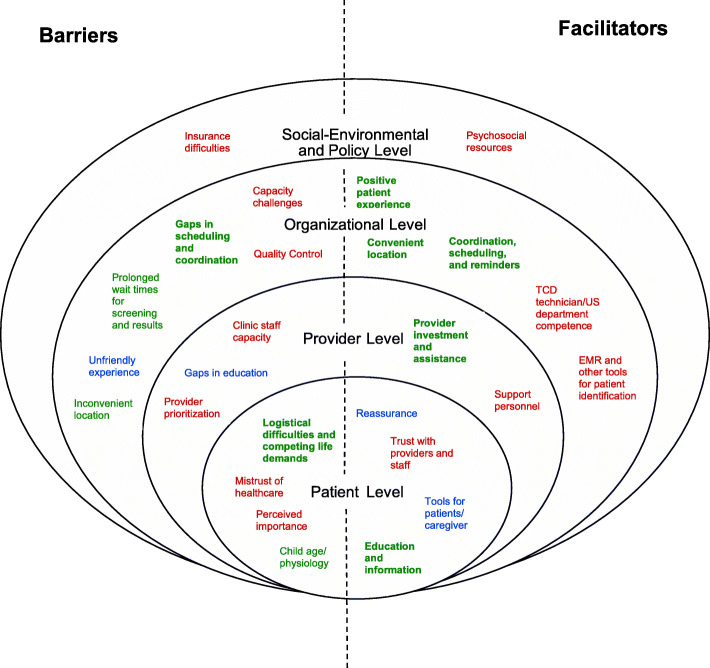
Themes by ecological level. Legend: predominant themes (text in bold), patient/caregiver report (text in blue), provider report (text in red), patient/caregiver and provider report (text in green)

Fourteen themes emerged representing barriers to TCD screening; 6 barrier themes were organizational level, 4 were patient level, 3 were provider-level, and 1 was social-environmental and policy level (Fig. 1). Two of the 14 barrier themes were predominant. Twelve themes emerged representing facilitators to TCD screening; 5 facilitator themes were organizational level, 4 were patient level, 2 were provider level, and 1 was social environmental and policy level. Five facilitator themes were predominant. Illustrative quotes for each predominant theme are presented in Table 3.

**Table 3:** Illustrative quotes for predominant themes

Theme	Illustrative quotes
**Barriers**	
Logistical difficulties and competing life demands	**Pt/CG 8**: “Just the transportation challenges that we experience to get there…” **Pr16**: “Most people are really trying to make it. It’s hard enough to do the day-to-day thing and you add in a medical disorder or work. It’s hard for them to follow through…the biggest issue is a lot of people just don’t remember their appointments.”
Gaps in scheduling and coordination	**Pt/CG10**: “So, definitely the scheduling time can be a little iffy because, like I said, they only have one person who does it.” **Pr12**: “If a patient misses their appointment with us and it’s the same day that they’re scheduled to have a TCD, we have a major problem with getting them rescheduled for the TCD.”
**Facilitators**	
Education and information	**Pt/CG10**: “You can never have too much information. Some people might not understand it like I got it. So, you know, definitely more education as far as that goes.” **Pr20**: “I have to adequately educate families. I have to make sure they know what I’m talking about…that they understand the importance.”
Positive patient experience	**Pt/CG16**: “They have a little screen where the kids can watch cartoons and relax…that’s the biggest thing…keeping them calm and putting them in a good place.”
Provider and staff investment and assistance	**Pt/CG19**: “I do think them making appointments is really helpful because when I try to make appointments at any other unit within the hospital, it’s always painful to do.” **Pr1**: “I actually just threatened [to radiology] that I myself was going to go and get training to do TCDs myself.”
Coordination, scheduling, and reminders	**Pr23**: “The resource that we have is phenomenal, in that the TCD person comes here and does it in one of our exam rooms. That’s our best resource.” **Pr15**: “We do all of the screening tests, visits, vaccinations…in one day as much as possible.”
Convenient location	**Pt/CG24**: “It’s in the same facility…they took us from the clinic to it. It wasn’t two or three minutes away.”

*Pt/CG* patient/caregiver participant, *Pr* provider participant

### Predominant barriers

#### Logistical difficulties and competing life demands (patient level)

Twenty participants described barriers in this theme; the majority were providers (*n* = 18). Providers frequently stated many patients missed repeated scheduled appointments resulting in downstream effects. Thus, patients could go years without screening and other departments, such as radiology, often were reluctant to reschedule for fear patients would not come in, resulting in unused appointment slots. There were also challenges with follow-up after completed screenings. Tracking was particularly concerning for patients with abnormal TCDs who needed either repeat TCD or CRCT. Providers reported concern over patients lost to care with missed appointments and attributed missed appointments to logistical issues, including transportation and limited resources. Family competing demands were another frequently mentioned cause for missed appointments, including conflicts with work schedules and school attendance and having other children in the home. Being unable to contact patients to schedule or reschedule appointments confounded other barriers. The barriers reported by 2 patient/caregiver participants in this theme corresponded with provider-reported barriers, including transportation challenges and forgetfulness associated with being a single parent with multiple children.

#### Gaps in scheduling and coordination (organizational level)

Of 18 participants who reported barriers in this theme, 7 were patients/caregivers and 11 were providers. Patients/caregivers and providers felt complicated processes for scheduling created a barrier to screening, particularly when TCD scheduling was separate from clinic scheduling. This problem was compounded when the initial appointment needed to be rescheduled and led to challenges with additional days of missed work and school. The problem of missed work and interruptions in daily life was exacerbated when patients/caregivers were unable to schedule the TCD appointment the same day as the routine clinic appointment. Similarly, providers expressed frustration with being unable to schedule same-day TCD screening when a patient was seen in the clinic who was overdue for TCD or in urgent need (e.g., required a follow-up screening for prior abnormal result). Providers also described issues with coordination amongst departments, particularly with radiology, where TCD screenings are typically conducted.

### Predominant facilitators

#### Education and information (patient level)

Patients/caregivers (*n* = 9) and providers (*n* = 6) emphasized the importance of patients/caregivers feeling educated and informed about TCD screening. Providers perceived patients/caregivers who understood the utility of TCD screening and the importance of the results were more likely to be adherent. Both groups of participants believed it necessary to educate the parent/caregiver and child about TCD screenings and stroke risk to help patients and families better understand the importance of screening. Patients/caregivers further discussed the benefit of radiology staff who are knowledgeable and communicative and explain the procedure as it is being done, while providers further discussed the need for repeated education to families and providing information from multiple sources and by multiple methods.

#### Positive patient experience (patient level)

Patients/caregivers (*n* = 9) more frequently discussed positive patient experience as a facilitator to TCD screenings than providers (*n* = 2). Patients/caregivers and providers mentioned the importance of resources that improve the child’s experience during a TCD, such as child life specialists and televisions for distraction. In addition, patient/caregiver participants mentioned keeping the child relaxed during the procedure and offering a reward for cooperation were facilitators. Patients/caregivers also described efforts made by parents toward making the child comfortable during the procedure, such as preparing the child the day before and offering an electronic device for distraction. According to patient/caregiver participants, the short duration of the TCD procedure was fundamental to facilitating the child’s cooperation.

#### Provider and staff investment and assistance (provider level)

Many participants noted that coordinating appointments with both radiology and the clinic could be challenging. Five patient/caregiver participants explicitly described sickle cell clinic staff making the TCD appointments for them as a facilitator to overcoming this challenge. Ten provider participants described additional efforts undertaken by sickle cell clinic staff and radiology staff as facilitators. Provider participants described nurses at sickle cell clinics who “call and beg” for same-day TCD screenings for patients in urgent need, such as those who were late for screening or needed a follow-up screening for a prior abnormal result. Efforts also included attempts by sickle cell clinic staff to resolve quality concerns by developing tracking systems to ensure TCDs are completed on schedule, and meeting weekly to discuss abnormal TCD results. Examples of radiology staff investment in obtaining TCD screenings included technicians who offer to travel to satellite clinics to conduct screenings, understand the importance of an abnormal result and immediately notify a provider, and are open to discussing training opportunities.

#### Coordination, scheduling, and reminders (organizational level)

This was the most commonly occurring facilitator theme among patient/caregiver participants (*n* = 19). Six providers contributed to this theme. Patients/caregivers and providers reported the TCD appointment scheduled the same day as a clinic appointment was a key facilitator to patients receiving screening. The use of reminders for appointments, made through various methods (phone, email, text, portal), was another key facilitator described by patients/caregivers and providers. Patients/caregivers and providers mentioned the importance of the clinic and radiology coordinating to schedule and conduct appointments. Some patients/caregivers reported involvement of the radiology department in coordinating appointments with clinic visits and sending reminders, while providers emphasized the importance of having a radiology department that allows flexibility in scheduling and utilization of unused TCD appointment slots.

#### Convenient location (organizational level)

Ten participants, 3 patient/caregivers, and 7 providers described the convenience of the TCD screening location as a facilitator. For some providers, this meant the capability to conduct TCD screenings in the clinic, which also allowed providers a high degree of control to capture patients lost to follow-up or late for screenings. Some providers also mentioned the benefit of conducting TCD screenings at satellite clinics, which prevents families from traveling to a more distant, central clinic for screenings. Patients/caregivers and providers described having the location where TCDs are conducted close to the clinic as a facilitator because families do not have to leave the building or only have a short walk between the clinic appointment and TCD appointment.

### Remediability assessment

Table 4 describes a list of interventions that were considered based on predominant barriers and facilitators. Our first step in this assessment was to ensure that there were viable interventions that would not only address the themes, but that would also improve the primary outcome of interest, rates of TCD implementation. During this initial process, the theme *Positive Patient Experience* was removed from further consideration. While patient experience is very important in terms of satisfaction with TCD and potentially with the quality of TCD, it was unclear whether addressing this facilitator would yield a significant impact on rates of TCDs when compared to the other predominant themes. Additionally, other themes were more evenly described by both patient/caregivers and providers and were therefore viewed to have the potential for a bigger impact. The remaining themes were considered remediable, and interventions to address each theme were evaluated based on cost, complexity, and adaptability.

**Table 4:** List of interventions considered and remediability assessment

Predominant barriers	Interventions considered	Cost	Complexity	Adaptability
Logistical difficulties and competing life demands	Coordination support	Salary or time spent from existing personnel assuming coordination responsibilities	Potential need to integrate new role/team member into clinic and disrupt work processes or flow	Sites indicated ability to create this role or hire new personnel with study support; sites indicated flexibility in how role is implemented
	Social worker	Salary or time spent from existing personnel assuming additional responsibilities	Potential need to integrate new role/team member into clinic; need to target several logistical challenges and competing life demands for families	Hiring processes may dictate role expectations at local hospitals that conflict with peripheral adaptability of intervention delivery
	New patient tracking software	Consulting cost with software developer; monetary cost of maintaining tool; cost of personnel’s time to use the tool	Tracking tool separate from electronic medical record/new process; multiple providers at a single site may need to learn the tool; potential disruption of workflow	Able to customize tracking process to meet local needs
	Transportation vouchers	Monetary cost of vouchers; personnel time to distribute vouchers	Range of transportation modes need to be accommodated (e.g., taxi, train, bus)	Depends on ease of using vouchers for specific transportation needs of patients
Gaps in scheduling and coordination	Coordination support	**	**	**
	New patient tracking software	**	**	**
Education and information	Educational materials and simplified explanation of TCD screening	Printing materials; consultant for branding and wording	Potential difficulty adopting a new process for education; multiple providers may complicate education	Uniform description of TCD can be used across sites and sites can modify peripheral information as needed (e.g., location); potential difficulty with language barriers
Provider and staff investment and assistance	Coordination support	**	**	**
	New patient tracking software	**	**	**
Coordination, scheduling, and reminders	Coordination support with reminders	**	Added complexity to role of coordinator if reminders are added; range of reminder methods; change in workflow processes	**
Convenient location	TCD ultrasound in clinic	TCD equipment and salary for trained technician	Variation in hospital policies for TCD process (e.g., location, billing); change in workflow processes; potentially high number of targeted units and people involved	Uncertain and potentially dependent on relationships with other hospital departments (e.g., radiology)

*TCD* transcranial Doppler

**Intervention described in cells above

One of the 2 predominant barrier themes was considered remediable and well suited to address via intervention for improving rates of TCD implementation: *Gaps in Scheduling and Coordination*. While some barriers within the theme *Logistical Difficulties and Competing Life Demands* could be addressed via intervention (e.g., issuing vouchers for transportation, tracking software to identify patients with missed appointments who need support), the variation in barriers was too vast to address with a single, cost-effective intervention. Effective coordination support was also felt to yield a higher likelihood of accommodating families’ competing life demands and related scheduling challenges. Two of the 3 predominant facilitator themes with the highest frequencies were remediable and suited to addressing via intervention: *Coordination*, *Scheduling*, *and Reminders* and *Education and Information*. The third highest-frequency predominant theme, *Provider and Staff Investment and Assistance*, was not considered well-suited to intervene upon because the heterogeneity in practice culture across sites made the development of a single intervention that could be applied across sites too complex; however, specific provider behaviors described during interviews were consistent with coordination and may be addressable via a coordination intervention and/or by tracking software. To this end, *Gaps in Scheduling and Coordination*/*Coordination*, *Scheduling and Reminders*, and *Education and Information* were determined to be well-suited for interventions for part 3 of the DISPLACE project. Finally, while *Convenient Location* was considered, the cost and complexity of co-locating TCD screening within the hematology clinic and simultaneously altering schedules and capacity for performing TCD across multiple departments were too high to make an intervention feasible.

## Discussion

This study was conducted to identify key barriers and facilitators to TCD screening for children with SCA to design the upcoming implementation study for DISPLACE. A notable strength of this study was that it gathered perspectives from a nationally representative sample of patients/caregivers and providers, which allowed a comprehensive analysis of factors across ecological levels. Findings support previous work examining barriers to TCD screening in SCA [15, 16, 18–26]; however, this study highlights several new and important points, including the complexity in barriers and facilitators interacting with one another at multiple ecological levels, the importance of care coordination, and the discrepancy in perceptions of barriers and facilitators between patients/families versus providers.

Two barrier and five facilitator themes emerged as predominant in this study; some are consistent with previous single-site and state-wide, claim-based studies. Specifically, prior studies supported the importance of patient/family adherence to scheduled appointments and the role of the provider in disseminating knowledge and improving ordering practices. Previous studies also noted organizational factors, such as co-locating TCD screening near the hematology clinic, as methods for improving TCD screening rates [15, 16, 18–26]. The current study used a more comprehensive approach to assess how factors influence and compound one another resulting in the novel findings described. For example, a key factor in the barrier *Gaps in Scheduling and Coordination* was rescheduling missed appointments. On the surface, this factor may not seem complex; however, the problem is affected by patient/caregiver life demands, such as missed work and school, provider tracking and identifying appointments that need to be rescheduled, and the organizational systems and capacity for rescheduling. Care coordination also emerged as a prominent theme (both as a barrier and a facilitator) not previously emphasized in the literature. Coordination could occur across multiple levels, including between patients, providers in both specialty care and radiology or other departments that provide TCD, clinic support staff, and schedulers. The use of multiple perspectives and the Multilevel Ecological Model of Health allowed us to capture this complexity, and the results emphasize the need for multi-level interventions to improve TCD screening rates.

Much was learned by comparing and merging patient/caregiver and provider perspectives. However, differences in perspectives between the groups were observed. Some barriers or facilitators were more frequently described by one group than the other. For example, *Logistical Difficulties and Competing Life Demands* was endorsed by many more providers than patient/caregivers while the reverse was observed with *Positive Patient Experience*. Notably, many patient/caregiver participants described few barriers to TCD screening. This finding is particularly important given the large number of logistical and family-related barriers described by providers, while only two patients/caregivers described barriers in this area. The discrepancy between patient/caregiver and provider perspectives was also noted in a previous qualitative study of caregiver perspectives on barriers to TCD screening in SCA, which found low knowledge and self-efficacy were more prominent factors for caregivers as opposed to practical barriers, such as lack of transportation, financial issues, and clinic hours in relation to adherence with TCD screening [21]. Results point to the importance of obtaining perspectives from both patients/caregivers and providers to inform intervention development.

As this study was conducted to inform intervention development for the upcoming implementation trial, it was critical to evaluate which barriers were addressable and which facilitators could be widely adopted via interventions. Some barriers or facilitators emerged as highly predominant but represented systems or social issues too widescale, complex, or costly to address via multi-site research study. Conversely, facilitators that could be adapted in different organizations and sustained over time were considered remediable and ideal for the next phase of the overall study.

### Limitations

The results of this study should be considered in the context of limitations. While purposive sampling was used to identify and recruit patients/caregivers, it was easier to recruit patients/caregivers with a history of more adherent behavior. The additional intention was required to recruit families who had been “less adherent” with slightly less participation from this group. However, the barriers and facilitators described by both adherent and less adherent families were consistent, including the finding that patients/caregivers did not feel there were substantial barriers to obtaining TCD screening. In addition, patient/caregiver participants were recruited from institutions participating in the DISPLACE project and were associated with academic institutions. The selection of CFIR constructs to frame assessment of remediability was guided by study priorities and time and budget constraints, but the exclusion of some CFIR constructs may have biased remediability assessment. Finally, due to time and budgetary constraints, we were not able to interview hospital administrators to obtain additional organizational perspectives.

## Conclusions

Results of this study highlight the challenges in bridging the research to practice gaps and underscore the difficulty in the communication of important health-related information. These barriers to communication are inherent in all disease populations but likely are exacerbated by negative social determinants of health. Findings emphasize the complexities of implementing change for even a single new clinical guideline. Although the NHLBI guidelines were published in 2014, it has been 20 years since the original STOP trial and implementation remains mediocre. Further, TCD screening rates remain far below the level needed to attain a “stroke-free generation” [15–18, 39]. Our next step for the DISPLACE project will be implementing interventions based on these findings to improve real-world TCD screening rates for children with SCA. Results may also inform clinical programs outside of the DISPLACE consortium in terms of the range of factors that should be considered when determining how to improve TCD screening rates in specialty care clinics.

## Supplementary Information

**Additional file 1.** DISPLACE Key Informant Interview Guide: Young Adults/Caregivers

**Additional file 2.** DISPLACE Key Informant Interview Guide: Providers

## Data Availability

The data used and analyzed during the current study are available from the corresponding author on reasonable request.

## Abbreviations

ASH: American Society of Hematology
CRCT: Chronic red cell transfusion
DISPLACE: Dissemination and Implementation of Stroke Prevention Looking at the Care Environment
SCA: Sickle cell anemia
STOP: Stroke Prevention Trial in Sickle Cell Anemia
STOP II: Optimizing Primary Stroke Prevention in Sickle Cell Anemia
TCD: Transcranial Doppler ultrasound
NHLBI: National Heart, Lung, and Blood Institute
U.S.: United States
