# Supplementary material for: Identified barriers and facilitators to stroke risk screening in children with sickle cell anemia: results from the DISPLACE consortium
Source: Implement Sci Commun. 2021 Aug 10;2:87. doi: 10.1186/s43058-021-00192-z (PMC8353775; doi:10.1186/s43058-021-00192-z)
Supplement: Supplementary file 1 — Additional file 1. DISPLACE Key Informant Interview Guide: Young Adults/Caregivers [file 43058_2021_192_MOESM1_ESM.docx]

**DISPLACE Key Informant Interview Guide: Young Adults/Caregivers**

1. **Healthcare Providers**

**“**The first questions are about the healthcare providers you/your child sees and the healthcare your child receives.”

- What types of healthcare providers do you/your child typically see?
- What are your thoughts about the care you/your child receives?
- How satisfied are you with the care you/your child receives?
- What would you change about the care you/your child receives?
- What types of things make it more difficult for you/your child to receive care? (insurance, transportation, location of clinic, etc.)
- What types of things help you/your child get the care you/he/she needs?

1. **Symptoms and Complications.**

“Great, thank you. Now I’d like to ask a few questions about your/your child’s sickle cell disease.”

- What symptoms do you/your child have from sickle cell disease? (offer examples of symptoms if needed)
- What do you usually do to help with these symptoms?
- Have you ever been told that you/your child has had complications from sickle cell disease, such as acute chest syndrome or stroke?
- If yes, please tell me about these complications.

1. **Transcranial Doppler Screening.**

“Thanks. I’d also like to ask you about Transcranial Doppler, or TCD screening.”

- Have you/your child received TCD screening?

If yes:

- - What was your experience like with TCD screening?
  - What made it difficult for you/your child to receive a TCD screening?
  - What helped you with obtaining your/your child’s TCD screening?
  - Was any follow up recommended after the TCD screening?

If no:

- - Have you heard of TCD screening?
  - (if applicable) Has anyone ever recommended a TCD screening for you/your child?
  - (if applicable) What happened that led to you/your child not receiving the screening?
  - (if applicable) What barriers have you experienced that kept you/your child from receiving a TCD? (such as insurance, transportation, or other barriers)
  - If a TCD screening was recommended for you/your child in the future, would you consider it? Why or why not?

1. **Chronic Red Cell Transfusion Therapy.**

“Okay, thank you. The next questions are about transfusions for sickle cell disease.”

- - Do you/your child receive red blood cell transfusions?

If yes:

- - Please tell me about your/your child’s experience with receiving blood transfusions. (how often, location, positive/negative experiences)
  - What makes it difficult to receive transfusions?
  - What helps you with transfusions?

If no:

- - Has anyone ever recommended you/your child receive a red blood cell transfusion?
  - Why do you think the transfusion was recommended?
  - What happened that led to not receiving a transfusion?
  - If red blood cell transfusions were recommended in the future, would you consider them? Why or why not?

**5. Developmental Assessment/Intervention**

“Great. Thank you. These last few questions have to do with how your child is doing in terms of his/her overall functioning. Sickle cell disease can sometimes cause problems with development, learning, and behavior in children. We want to better understand whether families have been offered certain types of services to improve these areas and how you feel about these services."

- Has your child ever received any type of developmental assessment or screening? [Clarify that these evaluations are typically done by a psychologist in a clinic or school and have various names - developmental evaluation, neuropsychological evaluation, neurocognitive evaluation, or psychoeducational evaluation]
- If yes:
  - Please tell me about your experience with your child receiving a developmental (or other term caregiver mentions) assessment (reason for evaluation, location, professional who completed, positive/negative experiences)
  - What made it difficult to obtain the evaluation?
  - What helped you to get the evaluation?
- If no:
  - Has a developmental assessment ever been recommended for your child?
  - Why do you think the assessment was recommended?
  - What happened that led your child to not receive the assessment?
  - If a developmental assessment was recommended for your child in the future, who you consider it? Why or why not?
- Has your child ever received intervention services related to development, learning, or behavior? (examples include speech/language therapy, occupational therapy, physical therapy, early intervention, school services, counseling, behavior therapy)
- If yes:
  - Please tell me about your experience with these services (reason for service, location, professional who provided the service, positive/negative experiences)
  - What made it difficult to obtain these services?
  - What helped you to get these services?
- If no:
  - Have these services ever been recommended for your child?
  - Why do you think the services were recommended?
  - What happened that led your child to not receive these services?
  - If these types of services were recommended for your child in the future, who you consider it? Why or why not?

“Thank you again for taking the time to talk with me today. Is there anything else you can think of that you’d like to share with us?”

*End interview, end recording*
