# Supplementary material for: Identified barriers and facilitators to stroke risk screening in children with sickle cell anemia: results from the DISPLACE consortium
Source: Implement Sci Commun. 2021 Aug 10;2:87. doi: 10.1186/s43058-021-00192-z (PMC8353775; doi:10.1186/s43058-021-00192-z)
Supplement: Supplementary file 2 — Additional file 2. DISPLACE Key Informant Interview Guide: Providers [file 43058_2021_192_MOESM2_ESM.docx]

**DISPLACE Key Informant Interview Guide: Providers**

1. **General experience.**

“I’d like to begin by asking you some general questions about your experiences as a health care provider.”

- Please describe the population of individuals with sickle cell disease you see in practice. For example, what are the approximate numbers of patients, age ranges, demographic and clinical characteristics, etc.?
- What are the most common reasons for patient visits?
- What are the greatest challenges you face in providing care to this population?

1. **Provider Self-Efficacy and Provider Support.**

“Great, thank you. Now I’d like to ask you a few questions about your comfort with providing care for individuals with sickle cell disease.”

- How sure are you that you are providing evidence-based care for individuals with sickle cell disease?
- What resources (websites, journals, other providers etc.) do you rely on when you have a question about providing care for individuals with sickle cell disease?
- Of the resources you typically use, which have you found to be most helpful and why?
- What challenges have you encountered to accessing/obtaining resources?

1. **Care Environment.**

“Thanks. In addition to learning about the resources you use, I’d also like to ask you about your experiences with the care environment, including specialty providers, reimbursement, and community resources.”

- Please describe your access to specialty providers for individuals with sickle cell disease. How often do you refer patients with sickle cell disease to a specialty provider? What factors influence your decision to make a referral?
- How would you describe your experiences with the referral process?
- What community-based resources are available to which you can refer your patients with sickle cell disease? What services do they provide?
- How would you describe your experiences with these community-based resources?
- What policy-level factors influence the care you provide to individuals with sickle cell disease? For example, what challenges are there to reimbursement for services?

1. **Transcranial Doppler Screening and Chronic Red Cell Transfusion therapy.**

“Thank you. Next, I’d like to ask you about your experiences with Transcranial Doppler (TCD) screening and chronic red cell transfusion (CRCT) therapy.”

- Please describe your familiarity with TCD screening for children with SCD.
- Do you recommend TCD screening for children with SCD?

If yes:

- Please describe the process at your organization for TCD screenings.
- What are the greatest challenges you face to TCD screening for children with SCD?
- What resources are most helpful for TCD screening for children with SCD?
- Once a TCD screening is completed, what is your usual follow up plan?

If no:

- Please tell me about the reasons for not recommending TCD screening.
- Please describe your familiarity with CRCT therapy for children with SCD.
- Do you recommend CRCT therapy for children with SCD?

If yes:

- What are the greatest challenges you face to CRCT therapy for children with SCD?
- What resources are most helpful for CRCT therapy for children with SCD?

If no:

- Please tell me about your reasons for not recommending CRCT therapy for children with SCD.

1. **Perceptions of Patient Experiences.**

“Wonderful, thank you very much. Now, I’d like to shift from talking about your experiences as a provider to your perceptions of patients’ experiences.”

- Please describe the typical healthcare utilization patterns among your patients with SCD. For example, how often do you see them in the office, how often do they visit the emergency department, how often are they hospitalized, etc.?
- In your experience, what would you say are the key challenges individuals with SCD face to receiving care from a provider?
- In your experience, what factors or resources best support individuals with SCD to receiving care from a provider?
- In your experience, what would you say are the key challenges individuals with SCD face to receiving TCD screening and, if applicable, CRCT?
- In your experience, what factors or resources best support individuals with SCD to receiving TCD screening, and if applicable, CRCT?
- Are there certain populations of individuals with sickle cell disease who seem to face greater challenges than others? If so, please describe them.

**6. Developmental-Behavioral Practice Patterns**

“Great. Finally, I’m going to ask you some questions about practice issues as they relate to developmental and behavioral concerns in children with sickle cell disease. We are defining development and behavior broadly to encompass concerns about developmental delays, concerns for learning or academic performance, and concerns for emotional and behavioral wellbeing (including mental health disorders) across childhood.”

- Do you use any formal practices to screen for developmental and behavioral concerns in your clinic? For example, do you use any structured screening instruments or questionnaires?
- Do you use any informal practices to screen for developmental and behavioral concerns? (e.g., asking about developmental milestones, asking about parent or school concerns)
- Please describe what services are available in your institution and geographical area if these concerns arise? (e.g., access to evaluation and intervention services, access to psychology, social work, developmental-behavioral pediatrics)
- In your experience, what would you say are the key challenges to identifying developmental and behavioral concerns in children with sickle cell disease?
- In your experience, what would you say are the key challenges to treating developmental and behavioral concerns in children with sickle cell disease?
- In your experience, what factors or resources help to identify developmental and behavioral concerns in children with sickle cell disease?
- In your experience, what factors or resources help to treat developmental and behavioral concerns in children with sickle cell disease?

“Thank you again for taking the time to speak with me today. Is there anything else you can think of that you’d like to share with us?”

*End interview, end recording*
